# Energy-Efficient Algorithm for Multicasting in Duty-Cycled Sensor Networks

**DOI:** 10.3390/s151229860

**Published:** 2015-12-11

**Authors:** Quan Chen, Siyao Cheng, Hong Gao, Jianzhong Li, Zhipeng Cai

**Affiliations:** 1 School of Computer Science and Technology, Harbin Institute of Technology, 92 West Dazhi Street, Harbin 150001, China; chenquan@hit.edu.cn (Q.C.); csy@hit.edu.cn (S.C.); lijzh@hit.edu.cn (J.L.); 2 Department of Computer Science, Georgia State University, Atlanta, GA 30303, USA; zcai@gsu.edu

**Keywords:** multicasting, energy optimization, power aware, Steiner tree, duty cycle, wireless sensor networks

## Abstract

Multicasting is a fundamental network service for one-to-many communications in wireless sensor networks. However, when the sensor nodes work in an asynchronous duty-cycled way, the sender may need to transmit the same message several times to one group of its neighboring nodes, which complicates the minimum energy multicasting problem. Thus, in this paper, we study the problem of minimum energy multicasting with adjusted power (the MEMAP problem) in the duty-cycled sensor networks, and we prove it to be NP-hard. To solve such a problem, the concept of an auxiliary graph is proposed to integrate the scheduling problem of the transmitting power and transmitting time slot and the constructing problem of the minimum multicast tree in MEMAP, and a greedy algorithm is proposed to construct such a graph. Based on the proposed auxiliary graph, an approximate scheduling and constructing algorithm with an approximation ratio of 4lnK is proposed, where *K* is the number of destination nodes. Finally, the theoretical analysis and experimental results verify the efficiency of the proposed algorithm in terms of the energy cost and transmission redundancy.

## 1. Introduction

In recent years, wireless sensor networks (WSNs) have been used in monitoring and retrieving sensory data from the physical world [1,2,3,4,5,6,7,8,9,10,11], which are usually expected to last over several months or years. Therefore, it is very necessary to design an energy conservation mechanism for WSNs to extend the network lifetime [12,13,14]. Thus, a schema of the duty-cycle is proposed in WSNs. According to the duty-cycle schema, each node switches between the active and the dormant state periodically; the period of the dormant state is much longer than that of the active state in order to save energy. According to the works in [15,16,17,18,19], the duty-cycled schema has high performance in terms of energy savings.

Multicasting is a fundamental component service for one-to-many communications in wireless sensor networks, such as to support data dissemination [20,21,22] for distributed data management and remote network configuration (e.g., [23,24,25]). Therefore, developing an energy-efficient multicast protocol is very meaningful in WSNs. Due to this consideration, the minimum energy multicasting (MEM) problem is proposed, which seeks to disseminate the messages from the source node to all of the destination nodes with minimum energy cost. Nowadays, the MEM problem has attracted extensive attentions from the research community, and it is studied in both nodes always-awake sensor networks [26,27,28,29,30,31,32] and duty-cycled sensor networks [33,34,35,36]. In the nodes always-awake sensor networks, where each node can deliver the massages to one group of its neighboring nodes by only one transmission, the MEM problem is proven to be NP-hard, and some approximation algorithms have been proposed [26,27,28,29,30,31,32].

However, in the duty-cycled sensor networks, the MEM problem becomes more complicated. Since the nodes can only receive the messages in the active state, the sender may need to transmit the same message several times to one group of its neighboring nodes. Therefore, the methods for the MEM problem in the nodes always-awake sensor networks are not suitable for duty-cycled sensor networks. In such networks, designing the minimum energy multicasting algorithm requires us not only to select appropriate forwarding nodes, but also to schedule the transmitting time slot optimally. Considering this, several methods are proposed by [33,34,35,36], which seek to minimize the transmission redundancy and the number of transmissions during multicasting. However, they all assumed the energy cost for all of the transmissions is equivalent and did not consider the case that the transmitting power of the sensor nodes can be adjusted. According to [37], the sensor nodes can transmit at six different power levels, which range from 1 MW to 100 MW. In this case, the senders need not only to choose their transmitting time slot intelligently, but also schedule their transmitting power optimally to construct the multicast tree.

Therefore, in this work, we study the problem of minimum energy multicasting with adjusted power (MEMAP) in duty-cycled sensor networks, and it is proven to be NP-hard. To solve such a problem, an approximate scheduling and constructing algorithm is proposed.

In summary, the contributions of the paper are as follows. 

(1) The MEMAP problem in the duty-cycled sensor networks is proposed to minimize the energy cost during multicasting, and its NP-hardness is proven.

(2) In order to solve the MEMAP problem, an auxiliary graph is defined, and a greedy algorithm is given to construct such a graph.

(3) Based on the proposed auxiliary graph, an approximation algorithm is proposed for the MEMAP problem, and its approximation ratio is proven to be 4lnK, where *K* is the number of destination nodes.

(4) Extensive simulations are carried out, which verify that the proposed algorithm has high performance in terms of energy cost. 

The rest of this work is organized as follows. Section 2 surveys the related work. In Section 3, we present the preliminaries, including the network model and the problem definition. Section 4 proposes the algorithm design in detail. Simulation results are discussed in Section 5. Section 6 concludes the paper.

## 2. Related Works

In recent years, there has been a tremendous amount of studies for the MEM problem in both nodes always-awake networks and duty-cycled sensor networks. In the nodes always-awake networks, the main works that studied the MEM problem are [26,27,28,29,30,31,32]. In [26], the author firstly studied the problem of constructing the minimum power broadcast/multicast tree in the wireless sensor network where each node can adjust its transmission power continuously, and three greedy heuristic algorithms were proposed. On the basis of [26], Wan *et al.* proved that the method in [26] has a linear approximation ratio, and then, they proposed several approximation algorithms with a constant approximation ratio for the min-power multicast routing problem [27]. In [28], the minimum energy broadcasting problem was proven to be NP-complete, and there is no polynomial algorithm with an approximation ratio better than Ω(logn) until NP=P. In [29], the author proposed a centralized approximation algorithm with at most 8lnK-times the optimum when the wireless nodes can adjust their transmitting power discretely. The minimum energy all-to-all multicasting problem was studied in [30], which tries to build a shared multicast tree to reduce the energy consumption. Recently, Qiu *et al.* [31] studied the minimum energy cooperative broadcasting problem where receivers can combat transmission errors by combing the received packets from different senders. Baghaie *et al.* [32] try to formulate the optimal tradeoff between the energy cost and broadcast latency in the cooperative communication scheme. However, these methods were not suitable for the duty-cycled sensor networks.

In duty-cycled sensor networks, the main works that studied the energy-efficient multicasting problem are [18,19,33,34,35,36]. In [18,19], Feng and Guo *et al.* proposed an opportunistic forwarding scheme for reliable flooding and broadcasting by considering unreliable links in duty-cycled sensor networks. Lai *et al.* [33] proposed a broadcasting protocol to achieve a better tradeoff between the broadcast latency and transmission redundancy in the duty-cycled sensor networks. In [34], the authors studied the minimum transmission problem for broadcasting, and they proposed a centralized algorithm with an approximation ratio of 3ln(Δ+1), where Δ denotes the maximum node degree in the network. However, they adopted a restricted duty-cycling model where there is only one active time slot existing in the working schedule of each node. In [35], Su *et al.* proposed two optimal algorithms for the minimum energy multicasting problem and the delay-bounded minimum energy multicasting problem when the number of destinations (e.g., *K*) is small in the multicast session. Han *et al.* [36] removed the limitation of the size of destinations and proposed a polynomial time complexity approximation algorithm with an approximation ratio of 6ρH(Δ+1)+2ρ, where H(.) denotes the harmonic number and *ρ* is the approximation ratio of the minimum Steiner tree problem [38,39]. However, they assumed the energy cost to be equivalent for all of the transmissions. Therefore, their problem is actually to minimize the number of transmissions in the multicast session, and their method is not suitable for the case when the transmitting power can be adjusted.

Due to the above limitations, we consider the problem of minimum energy multicasting with adjusted power in duty-cycled sensor networks, in which we need to not only schedule the transmitting power optimally to construct the multicast tree, but also choose the transmitting time slot for each node intelligently.

## 3. Models and Problem Definition

Before presenting our algorithms in detail, we depict the models used in this work and the formal definition of the MEMAP problem.

### 3.1. Network Model

We assumed a multihop duty-cycled sensor network G=(V,E), where *V* is the set of sensor nodes and *E* denotes the set of edges. There exists an edge between two sensor nodes if they are within each other’s transmission range. So as to conserve energy, each sensor node works between two states, e.g., the dormant state and the active state. In the dormant state, the sensor node turns off all of its functional models (*i.e.*, sensing the environment, sending and receiving packets) and just waits to be scheduled. The node switches between the active state and the dormant state periodically.

Let T denote one working cycle for each node, which is divided into multiple time slots with equal lengths. The length of each time slot (e.g., *τ*) can be determined according to [40] to guarantee that the data packet can de delivered to the neighbor successfully in one time slot. Since each sensor node has two states, all time slots in one working cycle, *i.e.*, {1,2,...,T/τ}, can be separated into two disjointed subsets for any u∈V. We let $W\left(u\right)=\left\{t_{u}^{1},t_{u}^{2},...,t_{u}^{k}\right\}$ denote the working schedule of node *u*, which contains all active time slots of *u*. Then, {1,2,...,T/τ}-W(u) contains all dormant time slots of *u*. As the same setting in [35], we assume $t_{u}^{1},t_{u}^{2},...t_{u}^{k}$ are consecutive in this paper, and they can be set up according to the requirement of coverage or connectivity [41]. Nodes can switch to the active state according to their working schedule or when they have packets to be sent, but they can only receive the packets when they are in the active state. To deliver the data, the sender can switch to the active state when the receiver wakes up. Additionally, node *u*’s duty cycle can be calculated as |W(u)|×τ/T.

In addition, we assumed that each sensor node is equipped with an omnidirectional antenna, and the transmission power of each sensor node can be adjustable. There are L power levels at each node, *i.e.*, $P=\;\{p_{1},p_{2},...p_{L}\}$, where $p_{i}\left(1\leq i\leq L\right)$ denotes the transmitting power of the *i*-th power level. Without loss of generality, *P* is sorted ascendingly, that is $p_{i}\leq p_{j}$ if 1≤i≤j≤L. For each neighboring node *v* of *u*, there exists a minimum transmitting power required to guarantee *v* is under the transmission range of *u*, and we assume that the transmission power is symmetric. Let the Euclidean distance between *u* and *v* be d(u,v). According to the path loss model, *u* can communicate with *v* with power level *l* only if:

(1) $$\frac{p_{l}/d{\left(u,v\right)}^{\alpha }}{N(1+\phi )}\geq \beta$$

where *α* is the pass loss exponent, *β* is the minimum SINR value (signal to interference plus noise ratio) to guarantee successful reception and N(1+ϕ) is the background noise. In these parameters, β>1 and *α* usually belong to [2,4].

### 3.2. The MEMAP Problem in Duty-Cycled Sensor Networks

Given a multicast request, which includes a source node *s* and a set of destination nodes D(D⊆V-{s}), the MEM problem in the nodes always-awake sensor networks is to construct a multicast tree that satisfies that: (1) it is rooted at the source node *s* and spanning all of the nodes in *D*; (2) the sum of the transmission power at the non-leaf nodes is minimized. This problem involves selecting the nodes to transmit the message and scheduling the transmitting power, as well.

However, multicasting in the duty-cycled sensor networks is quite different. According to the discussion in the above section, the working schedule of receivers decides the time slot for which the sender can transmit the packet. Then, in such a network, the broadcasting character can only be used when the receivers wake up simultaneously. In other words, as for a node *u* with multiple children in the multicast tree, it would need to transmit the same packet several times if the working schedules of all of the children are not overlapped. Therefore, the MEM problem in such a network not only involves selecting the transmitting nodes, but also the transmitting time slot and the transmitting power for each non-leaf node. We call the transmitting power and the transmitting time slot the transmitting schedule, which is defined as follows:

Definition 1. (Transmitting schedule) Given a node u∈V, a transmitting power $p(p\in \left\{p_{1},p_{2},...,p_{L}\right\})$ and a time slot t(t>0), then the transmitting schedule (u,p,t) means node *u* can transmit the packet at time slot *t* with transmitting power *p*.

By exploiting the definition of the transmitting schedule, the MEMAP problem in duty-cycled sensor networks is then to construct a multicast tree *T* and determine the transmitting schedules for each non-leaf node, while the total energy cost is minimized. Before we present the formal definition of the MEMAP problem, we need to give some notations here.

We use NB(u) to denote the set of neighboring nodes of *u* in *G*, which means the set of nodes *u* can communicate with the maximal transmitting power. As for an arbitrary multicast tree *T*, we use nl(T) to denote the set of non-leaf nodes in *T* and use fa(u) to denote *u*’s father and ch(u) to denote the set of *u*’s children for any node u∈T. Let (u,v) be a tree edge in *T*; then, *u* denotes the father node of *v*, and *v* is a child node of *u*.

In the following, we firstly give the definition of a feasible solution.

Definition 2. (Feasible solution) Given a source node *s* and a set of destination nodes $D=\;\{d_{1},d_{2}...d_{K}\}$, a multicast tree *T* and the transmitting schedules on multicast tree *T*, denoted by M(T), where M(T)={(u,p,t)|u∈nl(T)}. *T* and M(T) are called a feasible solution for the MEMAP problem if they satisfy the following conditions:

1. *T* is rooted at *s* and spans all of the nodes in *D*;
2. For any two nodes u∈nl(T), v∈ch(u), there exists a transmitting schedule (u,p,t)∈M(T), where *v* is under the transmission range of *u* with transmitting power *p* and t∈W(v).

According to the above definition, the definition of the MEMAP problem is presented as follows:

Input:

1. A duty-cycled sensor network G=(V,E);
2. A source node *s* and a set of destination nodes $D=\{d_{1},d_{2}...d_{K}\}$.

Output: A multicast tree *T* and the transmitting schedules on the multicast tree *T*, denoted by M(T). The multicast tree *T* and M(T) satisfy the following conditions:

1. *T* and M(T) are a feasible solution for the MEMAP problem;
2. For any feasible solution for the MEMAP problem, denoted by $T^{\prime }$ and $M(T^{\prime })$, we have $\sum_{\left(u^{\prime },p^{\prime },t^{\prime }\right)\in M\left(T^{\prime }\right)}p^{\prime }\geq \sum_{\left(u,p,t)\in M(T\right)}p$.

In the following, we will prove that the MEMAP problem in the duty-cycled sensor networks is NP-hard in Theorem 1.

**Theorem 1.**  *The* *MEMAP problem in the duty-cycled sensor network is NP-hard.*

**Proof.**  Let the working schedule of all of the nodes be the same, then the MEMAP problem in the duty-cycled sensor networks is converted to the MEM problem in the nodes always-awake sensor networks. Since the MEM problem in the nodes always-awake sensor networks is proven to be NP-hard by reducing the set cover problem to it, there is no polynomial-time algorithm with an approximation ratio better than Ω(logK) for it unless NP=P [28]. Therefore, the MEMAP problem is also NP-hard, as it is a general case of MEM problem. ☐

Based on Theorem 1, there is no polynomial time algorithm for the MEMAP problem unless NP=P, so that we study the approximate method to solve it, which is discussed in the next section.

## 4. Approximation Algorithms for MEMAP Problem

### 4.1. An Overview of the Proposed Algorithms

Since the above problem is NP-hard, we propose an approximate algorithm with an approximation ratio of 4lnK. The proposed algorithm mainly includes four steps. Firstly, we construct a weighted auxiliary graph based on the original graph *G*, which is used to integrate the transmitting power and transmitting time slot scheduling problem and the minimum multicast tree constructing problem. Secondly, We derive the MEMAP problem from the minimum node-weighted Steiner tree problem on the auxiliary graph and exploited approximation algorithms for the minimum node-weighted Steiner tree problem. Thirdly, we transform the obtained approximate minimum node-weighted tree *T* in the auxiliary graph into a valid multicast tree $T^{\prime }$, which can be mapped into a feasible solution for the MEMAP problem through a series of transformations. Finally, according to the valid multicast tree $T^{\prime }$ in the auxiliary graph, we obtain the multicast tree $T^{\prime \prime }$ on the original graph and the transmitting schedules for each non-leaf node.

### 4.2. The Auxiliary Graph

To clarify, G=(V,E) is referred to as the original graph, and u∈V is referred to as the original node. The first step of our approach is to transform the original graph into a weighted auxiliary graph $G^{\prime }$ to assist in building the node-weighted Steiner tree. Before we introduce the definition of the auxiliary graph, we need to give two concepts used in the auxiliary graph.

Generation node: For any node u∈V, we use $u_{g}$ to denote *u*’s generation node in the auxiliary graph $G^{\prime }$.

Schedule node: For any node u∈V, we use $u_{s}$ to denote *u*’s schedule node in the auxiliary graph $G^{\prime }$. Each schedule node $u_{s}$ owns three properties, *i.e.*, ($u_{s}.g$, $u_{s}.p$, $u_{s}.t$), which denote its corresponding generation node, transmitting power and transmitting time slot, respectively. For any node u∈V, we call $u_{g}$ the corresponding generation node of $u_{s}$, and $u_{s}$ is derived from $u_{g}$ in this paper. Obviously, we can get $u_{s}.g=u_{g}$.

Definition 3. (Auxiliary graph) Given a duty-cycled sensor network G=(V,E), its auxiliary graph $G^{\prime }=\left(V^{\prime },E^{\prime }\right)$ denotes the graph containing the scheduling information, where $V^{\prime }$ and $E^{\prime }$ denote the set of nodes and edges. $V^{\prime }$ and $E^{\prime }$ are constructed as follows:

(i) Initially, $V^{\prime }=\emptyset$, $E^{\prime }=Ø$;
(ii) For each node u∈V, we add a generation node $u_{g}$ in the auxiliary graph $G^{\prime }$, *i.e.*, $V^{\prime }=\left\{u_{g}\;|u\in V\right\}$;
(iii) For each node u∈V, we then add L schedule nodes in $G^{\prime }$, *i.e.*, $u_{s1},u_{s2},...u_{sL}$, where the three properties of $u_{sl}\left(1\leq l\leq L\right)$ are set as $u_{sl}.g=u_{g}$, $u_{sl}.p=p_{l}$ and $u_{sl}.t=0$, respectively. It is to be noticed that the transmitting time slot of $u_{s}$ is initialized as zero, and we will introduce the method to determine $u_{sl}.t$ later. Let the set of all of the schedule nodes of *u* be denoted by Υ(u). Then, we can have $V^{\prime }=V^{\prime }\underset{u\in V}{⋃}\Upsilon \left(u\right)$.
(iv) For each node u∈V, we create an edge between $u_{g}$ and each schedule node $u_{s}\in \Upsilon \left(u\right)$, which means node *u* can transmit with power $u_{s}.p$ on time slot $u_{s}.t$. Let $E_{u}^{\prime }=\left\{\left(u_{g},u_{s}\right)|u_{s}\in \Upsilon \left(u\right)\right\}$, then we can have $E^{\prime }=\underset{u\in V}{⋃}E_{u}^{\prime }$;
(v) Let *v* be a neighboring node of *u* in the original graph G, and $v_{g}$ is the generation node of *v*. For any schedule node $u_{s}\in \Upsilon \left(u\right)$, we add an edge $\left(u_{s},v_{g}\right)$ in $G^{\prime }$ if only if *v* is under the transmission range of *u* with transmitting power $u_{s}.p$, and we use $R(u_{s})$ to denote the set of such nodes *v* of $u_{s}$. After then, $E^{\prime }$ can be updated as $E^{\prime }=E^{\prime }\underset{u\in V}{⋃}\left\{\underset{u_{s}\in \Upsilon \left(u\right)}{⋃}E_{u_{s}}^{\prime }\right\}$, where $E_{u_{s}}^{\prime }=\left\{\left(u_{s},v_{g}\right)|v\in R\left(u_{s}\right)\right\}$.

**Figure 1:**
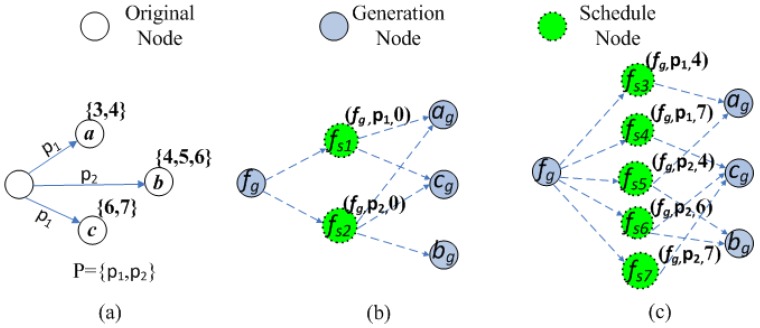
An example of constructing the auxiliary graph. (**a**) The original graph; (**b**)The intermediate auxiliary graph; (**c**) The final auxiliary graph.

As the example in Figure 1, there is an original graph in Figure 1a, where the number in the braces denotes the working schedules. There are two power levels $\left\{p_{1},p_{2}\right\}$ for each node, and $p_{1}<p_{2}$. As for the forwarder *f*, it can reach *a* and *c* with transmission power $p_{1}$ and *a*, *b*, *c* with transmission power $p_{2}$. As for the above original graph, we do as follows according to Definition 3, where the result is shown in Figure 1b. Firstly, the generation nodes are created for each original node, *i.e.*, the blue nodes in Figure 1b, which are $f_{g}$, $a_{g}$, $b_{g}$ and $c_{g}$, respectively. Then, we create two schedule nodes $f_{s1}$ and $f_{s2}$ with transmitting power $p_{1}$ and $p_{2}$, respectively, and their three properties are $\left(f_{g},p_{1},0\right)$ and $\left(f_{g},p_{2},0\right)$. According to the above discussion, we connect $a_{g}$ and $c_{g}$ to $f_{s1}$ and connect $a_{g}$, $b_{g}$ and $c_{g}$ to $f_{s2}$.

In the following, we will introduce the transmitting time slot-determining algorithm to determine the transmitting time slot for each schedule node $u_{s}$ in the constructed auxiliary graph $G^{\prime }$. Before that, we need to give some notations.

Let $start\left(u\right)=min\{t_{u}\;|t_{u}\in W\left(u\right)\}$ and $end\left(u\right)=max\{t_{u}\;|t_{u}\in W\left(u\right)\}$ denote the first active time slot and the last active time slot of node *u*, respectively. Since the sensor nodes can only receive the data message when it is in the active state, the transmitting time slot must be assigned as the slot that all of its reaching nodes are active. For a schedule node $u_{s}$ and all of the reaching nodes in $R(u_{s})$, it may require several slots to deliver the message to all of them. For this case, the schedule node $u_{s}$ may be split into multiple schedule nodes with the same generation node and transmitting power (e.g., $u_{s}.g$ and $u_{s}.p$), but different transmitting time slots (e.g., $u_{s}.t$). In addition, for any transmission schedule (u,p,t) in the optimal schedule, we should find a corresponding schedule node in the auxiliary graph.

For any schedule node $u_{s}$ in Definition 3, the transmitting time slot-determining algorithm exploits a greedy strategy, which mainly works as follows:

Firstly, all of the nodes in $R(u_{s})$ are sorted by their ending time slots in ascending order.

Secondly, we greedily choose the last active time slot of the first node in $R(u_{s})$ to create a new schedule node. Let $v_{1}$ be such a node, and then, we create a new schedule node $u_{sL+1}$ and set $u_{sL+1}.t=end\left(v_{1}\right)$. For any node $v\in R(u_{s})$, we connect it to the new schedule node $u_{sL+1}$ if $start\left(v\right)\leq u_{sL+1}.t$. After that, we remove $v_{1}$ from $R(u_{s})$. For the nodes left in $R(u_{s})$, we repeat the same procedure, until all of the nodes in $R(u_{s})$ are handled.

Finally, since the original schedule node $u_{s}$ will not be used afterwards, we delete it from $G^{\prime }$ directly. The detailed procedure of the transmitting time slot-determining algorithm is shown in Algorithm 1.
**Algorithm 1** Transmitting time slot-determining algorithm.**Input:** A schedule node $u_{s}$, the set of its reaching nodes $R(u_{s})$; **Output:** The set of splitting schedule nodes $u_{sj}$ and its transmitting time slot $u_{sj}.t$;
1:Sorting the nodes in $R(u_{s})$ according to their last active time slot;2:j←L+1;3:**while** $R(u_{s})$ is not empty **do**4:  $v_{1}\leftarrow$ the first node in $R(u_{s})$;5:  Create a new schedule node $u_{sj}$ identical to $u_{s}$;6:  $u_{sj}.t\leftarrow end\left(v_{1}\right)$;7:  Add edge $\left(u_{g},u_{sj}\right)$ and $\left(u_{sj},v_{1}\right)$ in $G^{\prime }$;8:  **for** i=2 to $\left|R(u_{s})\right|$ **do**9:      $v_{i}\leftarrow$ the *i*-th node in $R(u_{s})$10:      **if** $start\left(v_{i}\right)\leq u_{sj}.t$ **then**11:            add edge $\left(u_{sj},v_{i}\right)$;12:      **else**13:            break;14:      **end if**15:  **end for**16:  j←j+1;17:  Remove $v_{1}$ from $R(u_{s})$;18:**end while**19:Delete $u_{s}$ from $G^{\prime }$;20:**return** the set of splitting schedule nodes of $u_{s}$;


As shown in the above example, since the working schedules of nodes *a* and *c* are not overlapped, we split the schedule node $f_{s1}$ into two schedule nodes $f_{s3}$ and $f_{s4}$ to connect to $a_{g}$ and $c_{g}$, respectively. The transmitting time slot for $f_{s3}$ and $f_{s4}$ are set as 4 and 7 respectively, since end(a)=4 and end(c)=7. As for the schedule node $f_{s2}$, its reaching node $R\left(f_{s2}\right)=\left\{a,b,c\right\}$. We first choose the minimal last active time slot from $\left\{end\left(v\right)|v\in R\left(f_{s2}\right)\right\}$, e.g., end(a)=4, to create a new schedule node $f_{s5}$ with $f_{s5}.t=4$. Then, we connect $f_{s5}$ to node $a_{g}$ and $b_{g}$. After that, we remove *a* from $R(f_{s2})$. For the rest of nodes in $R(f_{s2})$, we do similarly and choose Time Slot 6 to create a new schedule node to connect node $b_{g}$ and node $c_{g}$. The complete auxiliary graph is shown in Figure 1c, and the three properties for each schedule node are shown in the brackets above the node.

Through the above procedure, we can see that the original schedule node is “split” into several schedule nodes with different transmitting time slots. The above greedy strategy can guarantee that for any transmission schedule (u,p,t) in the optimal solution, we can find a corresponding schedule node in the auxiliary graph, which is shown in Theorem 2.

**Theorem 2.**  *Assuming* $T_{opt}$ *and* $M_{opt}\left(T_{opt}\right)$ *are the optimal multicast tree and its transmitting schedules. Then, for any* $\left(u,p,t\right)\in M_{opt}\left(T_{opt}\right)$, *we can find a schedule node in the auxiliary graph* $G^{\prime }$.

**Proof.**  Let Ch(u,p,t) denote the set of the children of *u* in the multicast tree $T_{opt}$ where *u* can communicate with at time *t* by transmitting power *p*, and *v* is the node of the minimum last active time slot in Ch(u,p,t). In the following, we will prove this from two aspects.

(1) We will firstly prove that all of the working schedules of nodes in Ch(u,p,t) contain the time slot end(v). Assuming there is at least one node whose working schedule does not contain time slot end(v), since the working schedules are consecutive, then there must exist a node $v^{\prime }\left(v^{\prime }\neq v⋀v^{\prime }\in Ch\left(u,p,t\right)\right)$ that $end\left(v^{\prime }\right)\;<\;end\left(v\right)$ or $start\left(v^{\prime }\right)>end\left(v\right)$. As *v* denotes the node with the minimum last active time slot in Ch(u,p,t), so we have $end\left(v^{\prime }\right)\geq end\left(v\right)$. In addition, according to the definition of the MEMAP problem, the working schedules of all of the nodes in Ch(u,p,t) are overlapped, and all contain time slot *t*. Then, we can have t≤end(v) and $start(v^{\prime })\leq t$, which result in $start\left(v^{\prime }\right)\leq end\left(v\right)$. Combining the two reasons, we can get $end\left(v^{\prime }\right)\geq end\left(v\right)$ and $start\left(v^{\prime }\right)\leq end\left(v\right)$, which contradicts that $end\left(v^{\prime }\right)<end\left(v\right)$ or $start\left(v^{\prime }\right)>end\left(v\right)$. Therefore, all of the working schedules of nodes in Ch(u,p,t) contain the time slot end(v).

(2) Now, we will prove that the schedule node $u_{s}$ is the target node in two cases:

- If t=end(v), $u_{s}$ is the correspondent schedule node obviously.
- If t<end(v), since all of the working schedule of the nodes in Ch(u,p,t) contains the time slot end(v), so (u,p,end(v)) is also a feasible schedule that *u* can communicate with all of the nodes in Ch(u,p,t) with transmitting power *p* at time slot end(v). In this case, we can just map the transmitting schedule (u,p,t) to the schedule node $u_{s}$, as well.

Combing the above two reasons, the theorem is proven. ☐

So far, the auxiliary graph has been constructed. We can find that $V^{\prime }$ in the auxiliary graph can be partitioned into two subsets $V_{g}$ and $V_{s}$, where $V_{g}$ is the set of all generation nodes and $V_{s}$ is the set of all of the schedule nodes. In order to exploit the node-weighted Steiner tree algorithm, we set the weight of each generation node $u_{g}$ as $w(u_{g})=0$ and set the weight of each schedule node $u_{s}$ as $w\left(u_{s}\right)=u_{s}.p$.

The size of the auxiliary graph is analyzed in Theorem 3.

**Theorem 3.**  *The number of nodes and edges in the auxiliary graph* $G^{\prime }$ *are at most* n+n×L×Δ *and* (1+Δ)×(n×L×Δ), *respectively, where* Δ *denotes the maximum degree and* n=|V| *denotes the number of nodes in the original graph.*

**Proof.**  Firstly, as in Definition 3, for each generation node $u_{g}$ in the auxiliary graph $G^{\prime }$, there is only one schedule node $u_{s}$ with power level *l* in Υ(u), where Υ(u) denotes all of the schedule nodes of *u*. Then, the schedule node $u_{s}$ is split into several schedule nodes with power level *l* after executing the transmitting time slot-determining algorithm. Let $\Upsilon (u_{s},l)$ denote the set of schedule nodes derived from $u_{g}$, and its power level is *l*. In the worst case, we can have $|\Upsilon \left(u_{s},l\right)|=|NB\left(u\right)|\leq \Delta$. Then, for each node u∈V, we can have:

(2) $$\left|\Upsilon \left(u\right)\right|=\sum_{1}^{L}\left|\Upsilon (u_{s},l)\right|\leq \sum_{1}^{L}\left|NB(u)\right|\leq L\times \Delta$$

Thus, according to Equation (2), the total number of nodes in the auxiliary graph $G^{\prime }$ can be calculated as:

(3) $$|V^{\prime }\left|=\right|V_{g}\left|+\right|V_{s}\left|=\right|V_{g}|+\sum_{u\in V}\left|\Upsilon (u)\right|\leq n+n\times L\times \Delta$$

where $|V_{g}|=|V|=n$ denotes the number of nodes in the original graph.

Secondly, according to Definition 3 and Algorithm 1, for each generation node $u_{g}$, there exists an edge between $u_{g}$ and $u_{s}\left(u_{s}\in \Upsilon \left(u\right)\right)$. For each schedule node $u_{s}\in \Upsilon \left(u\right)$, there exists at most |NB(u)| edges from $u_{s}$ to its neighboring generation nodes. Then, we can have:

(4) $$\begin{array}{c} |E^{\prime }|=\sum_{u\in V}|E_{u}^{\prime }|+|\underset{u\in V}{⋃}\left\{\underset{u_{s}\in \Upsilon \left(u\right)}{⋃}E_{u_{s}}^{\prime }\right\}|\leq \sum_{u\in V}\left|\Upsilon (u)\right|+\sum_{u\in V}(|\Upsilon \left(u\right)\left|\times \Delta \right)\leq \left(1+\Delta \right)\times \left(n\times L\times \Delta \right) \end{array}$$

☐

As we can see, compared to the original graph, the nodes in the auxiliary graph increased L×Δ times and the edges increased $L\times \Delta^{2}$ times. Since L and Δ are usually constant, the size of the auxiliary graph is controlled. In addition, we can notice that the auxiliary graph $G^{\prime }$ has the following properties:

(1) Given two nodes in $G^{\prime }$, there are no edges between them if they are both schedule nodes or generation nodes. In other words, the neighbors of a schedule node are all generation nodes, and the neighbors of a generation node are all schedule nodes. Two generation nodes are connected through a schedule node.

(2) $R\left(u_{si}\right)\subseteq R\left(u_{sj}\right)$, if $1\leq u_{si}.p\leq u_{sj}.p\leq L$ and $u_{si}.t=u_{sj}.t$, where $u_{si}$ and $u_{sj}$ are two schedule nodes derived from the same generation node $u_{g}$.

(3) For any two generation nodes $u_{g}$ and $v_{g}$, which are connected to a same schedule node $w_{s}$, then the working schedules of node *u* are overlapped with *v*, which means they can receive the packet simultaneously.

### 4.3. Minimum Node-Weighted Steiner Tree

Given a multicast request that includes a source node *s* and a set of destination nodes *D* in the duty-cycled sensor network *G*, let $s_{g}$ be the generation node of *s* in the auxiliary graph $G^{\prime }$, and $D_{g}=\left\{d_{1g},d_{2g},...,d_{Kg}\right\}$ are the set of the generation nodes of the destination nodes. Now, our objective is to find a minimum node-weighted Steiner tree in $G^{\prime }$, which is rooted at $s_{g}$ and spanning all of the nodes in $D_{g}$. The minimum node-weighted Steiner tree was used to help us obtain a feasible solution for the MEMAP problem, which includes a multicast tree *T* and the transmitting schedules M(T).

However, it is unlikely to have a polynomial-time algorithm to find such a minimum node-weighted tree in the auxiliary graph $G^{\prime }$ unless P=NP. Thus, a approximation algorithm [38] is exploited to obtain the near optimal minimum node-weighted Steiner tree with approximation ratio of 2lnK.

Let $T_{ag}^{app}$ denote the obtained approximate minimum node-weighted Steiner tree in the auxiliary graph $G^{\prime }$. In the following, we will introduce the method to map $T_{ag}^{app}$ to a feasible solution for the MEMAP problem, which is guaranteed by the following theorem.

**Theorem 4.**  *Let* $T_{ag}$ *be a node-weighted Steiner tree, which is rooted at* $s_{g}$ *and spans all of the nodes in* $D_{g}$ *in the auxiliary graph* $G^{\prime }$; $T_{ag}$ *can be mapped to a feasible solution for the MEMAP problem if it satisfies the following conditions:*

*1*. *For any leaf node* $i\in T_{ag}$, *i must be a generation node in* $D_{g}$;
*2*. *For each schedule node* $u_{s}$ *in the node-weighted Steiner tree* $T_{ag}$, *there exists a generation node* $u_{g}$ *in* $T_{ag}$, *where* $u_{g}$ *is the corresponding generation node of* $u_{s}$;
*3*. *For any non-leaf generation node* $u_{g}$ *in the tree* $T_{ag}$ *and any node* $i\in ch(u_{g})$, *node i must be a schedule node derived from* $u_{g}$.

**Proof.**  In the following, we will prove the theorem by constructing a feasible solution for the MEMAP problem with the node-weighted Steiner tree $T_{ag}$.

Firstly, the multicast tree *T* in the original graph is constructed by the following three steps:

Step 1. For any schedule node in $T_{ag}$, we create an edge between its father and all of its child nodes;

Step 2. Remove all of the schedule nodes from $T_{ag}$;

Step 3. Replace all of the generation nodes with their original nodes.

As we can see, for any tree edge (fa(u),u)∈T, there exists a schedule node between fa(u) and *u* in the node-weighted Steiner tree $T_{ag}$, which means that fa(u) can communicate with *u* with a certain power. Additionally, $s_{g}$ and $D_{g}$ are all in $T_{ag}$, then *s* and *D* are in *T* accordingly. Therefore, the source node *s* can deliver the messages to all of the destination nodes by the tree *T*.

Secondly, we will show how to determine the transmitting schedules for *T*, *i.e.*, M(T). For each node *u* in *T*, $u_{g}$ is its corresponding generation node in $T_{ag}$. Then, for each schedule node $u_{s}\in ch\left(u_{g}\right)$ in $T_{ag}$, we add a transmitting schedule $\left(u,u_{s}.p,u_{s}.t\right)$ in M(T). Since for each non-leaf node *u* and its child node v∈T, there exists a schedule node between them, then we can have a transmitting schedule $\left(u,u_{s}.p,u_{s}.t\right)$ with which *u* can communicate with *v* with transmitting power $u_{s}.p$ at time slot $u_{s}.t$.

Combining the above two reasons and Definition 2, a feasible solution for the MEMAP problem is obtained. The theorem is proven. ☐

In this paper, we call a node-weighted Steiner tree a valid multicast tree if it satisfies the three conditions in Theorem 4.

**Figure 2:**
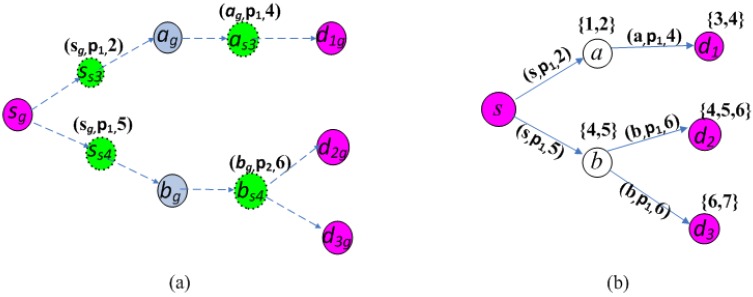
An example of a valid multicast tree.(**a**) A valid multicast tree; (**b**) The corresponding multicast tree and transmitting schedules.

As the example shown in Figure 2, there is a calculated Steiner tree in Figure 2a, where the pink node denotes the source node and the destination nodes. We can find that the tree in Figure 2a is a valid multicast tree since it satisfies the conditions in Theorem 4. According to the above method, then we can obtain the corresponding multicast tree and transmitting schedules on the original graph. The results are shown in Figure 2b, where the three tuple along the links denotes a transmitting schedule.

### 4.4. Constructing a Valid Multicast Tree

However, the obtained approximate minimum node-weighted Steiner tree $T_{ag}^{app}$ on the auxiliary graph $G^{\prime }$ may not satisfy the three conditions in Theorem 4. There exist three violations.

(a) Violation 1. $T_{ag}^{app}$ contains a leaf node *i*, and *i* does not belong to $D_{g}$. This violets Condition 1 in Theorem 4.
(b) Violation 2. $T_{ag}^{app}$ contains a schedule node, which is not derived from any of its neighboring generation nodes in the tree $T_{ag}^{app}$. This violets Condition 2 in Theorem 4.
(c) Violation 3. $T_{ag}^{app}$ contains a generation node $u_{g}$ ($u_{g}\neq s_{g}$), but $u_{g}$ cannot be reachable by the source node $s_{g}$, which means that there exist the tree edges $\left(v_{g},u_{s}\right)$ and $\left(u_{s},u_{g}\right)$ in $T_{ag}^{app}$, where $u_{s}$ is the father node of $u_{g}$ and a child node of $v_{g}$. This violets Condition 3 in Theorem 4.

In order to eliminate the three violations in the approximate minimum node-weighted Steiner tree $T_{ag}^{app}$, we introduce three correcting operations as follows.

For Violation 1: For any leaf node *i* that does not belong to $D_{g}$, we just delete it from $T_{ag}^{app}$. This procedure continues until all of the leaf nodes satisfy Condition 1 in Theorem 4.

For Violation 2: Assume $u_{s}$ is the schedule node, which is not derived from any of its neighboring generation nodes. Let $u_{g}$ be the generation node of $u_{s}$, then we do as follows:

1. if $u_{g}\notin T_{ag}^{app}$, then the generation node $u_{g}$ and the edge $\left(u_{g},u_{s}\right)$ are added in the tree $T_{ag}^{app}$;
2. if $u_{g}\in T_{ag}^{app}$, but $u_{g}\notin NB\left(u_{s}\right)$, where $NB(u_{s})$ denotes the set of neighbors of $u_{s}$ in the tree $T_{ag}^{app}$; in this case, we delete the tree edge $\left(fa\left(u_{s}\right),u_{s}\right)$ from $T_{ag}^{app}$ firstly, and then, we would add the tree edge $\left(u_{g},u_{s}\right)$ in the current tree $T_{ag}^{app}$.

For Violation 3: This correcting operation is done by checking all of the nodes in the tree $T_{ag}^{app}$ through a breadth-first search. All of the nodes in the tree have two states, e.g., “uncheck” and “checked” states. Let the queue *Q* store the set of current nodes needed to be checked. Initially, all of the nodes in $T_{ag}^{app}$ are marked “uncheck”, and the root $s_{g}$ is pushed into *Q*. The correcting process works as follows:

Let the first node in *Q* be *i*; we first marked node *i* “checked” and pop it from *Q*. Then, we handle *i* according to the following two cases:

Case 1: *i* is a generation node. Then, for each schedule node $j_{s}\in ch\left(i\right)$, we do as follows:

1. If $j_{s}$ is not derived from *i*, let $j_{g}$ denote the corresponding generation node of $j_{s}$. We then check for any schedule node $c_{s}\left(c_{s}\in ch\left(i\right)\right)$, whether there exists an edge between $c_{s}$ and $j_{g}$ in the auxiliary graph $G^{\prime }$. If yes, add an edge $\left(c_{s},j_{g}\right)$ in $T_{ag}^{app}$. Otherwise, we choose the schedule node $c_{s}=argmin\left\{w\left(i_{s}\right)|i_{s}\in \Upsilon \left(i\right)⋀\left(i_{s},j_{g}\right)\in E^{\prime }\right\}$ to add into the current tree $T_{ag}^{app}$, and then, the tree edges $\left(i,c_{s}\right)$ and $\left(c_{s},j_{g}\right)$ are added. After that, we delete the tree edge $\left(i,j_{s}\right)$ from $T_{ag}^{app}$. Finally, the new added schedule node $c_{s}$ is pushed into *Q*.
2. If $j_{s}$ is derived from *i*, we just push it into Q;

Case 2: *i* is a schedule node. Then, for any generation node $u_{g}\in ch\left(i\right)$, we just push it into *Q* for the following computing.

The correcting operation for Violation 3 ends when *Q* is empty, and all of the nodes in $T_{ag}$ are marked “checked”.

In the following, we will prove that the tree after the above three correcting operations can satisfy the three conditions in Theorem 4, which means it is a valid multicast tree.

**Theorem 5.**  *Let the tree after the three correcting operations be* $T_{ag}^{c}$; *then*, $T_{ag}^{c}$ *is a valid multicast tree.*

**Proof.**  In order to guarantee the correctness of Theorem 5, we just need to prove that the above three operations can eliminate the violations successfully, that is the three conditions in Theorem 4 are satisfied.

As for Condition 1 (for any leaf node $i\in T_{ag}^{c}$, *i* must be a generation node in $D_{g}$), according to correcting Operation 1, any leaf node *i* that is not a generation node in $D_{g}$ is pruned. Then, Condition 1 is satisfied.

As for Condition 2 (for each schedule node $u_{s}$ in the node-weighted Steiner tree $T_{ag}^{c}$, there exists a generation node $u_{g}$ in $T_{ag}^{c}$, where $u_{g}$ is the corresponding generation node of $u_{s}$), according to correcting Operation 2, for any schedule node $u_{s}$ in $T_{ag}^{c}$, its generation node $u_{g}$ and the tree edge $\left(u_{g},u_{s}\right)$ are added in the tree. Then, Condition 2 is also satisfied.

As for condition 3 (for any non-leaf generation node $u_{g}$ in the tree $T_{ag}^{c}$, then for any node, $i\in ch(u_{g})$ must be a schedule node derived from $u_{g}$), according to correcting Operation 3, for each generation node *i* in $T_{ag}^{c}$ and any schedule node $j_{s}$ that is not derived from *i*, we delete the tree edge $\left(i,j_{s}\right)$ in the obtained approximate minimum node-weighted Steiner tree and add a schedule node $i_{s}$, which is chosen to connect to $j_{s}$’s corresponding generation node $j_{g}$. Obviously, Condition 3 is satisfied.

Combining the above three reasons, the theorem is proven. ☐

After the approximate minimum node-weighted Steiner tree $T_{ag}^{app}$ has been corrected to a valid multicast tree in the auxiliary graph, then we can transform it into a feasible solution for the MEMAP problem according to Theorem 4. So far, the complete approximate scheduling and constructing algorithms have all been introduced, which is shown in Algorithm 2.
**Algorithm 2** Approximate scheduling and constructing algorithm.**Input:** A duty-cycled network *G*, a source node *s* and a set of destination nodes *D*; **Output:** A multicast tree *T* and the set of transmitting schedules M(T) for the multicast tree *T*;
1:Construct the auxiliary graph $G^{\prime }$ according to Definition 3;2:**for all** schedule node $u_{s}$ in $G^{\prime }$ **do**3:  Call Algorithm 1 to determine the transmitting time slot for each schedule node;4:**end for**5:Call the Steiner tree algorithm to get a multicast tree $T_{ag}^{app}$ on $G^{\prime }$;6:Correct $T_{ag}^{app}$ to a valid multicast tree $T_{ag}^{c}$ on $G^{\prime }$ by using the three correction operations in Section 4.4;7:Map the valid multicast tree $T_{ag}^{c}$ into a feasible solution for MEMAP using the method in Theorem 4, including the multicast tree *T* and the set of transmitting schedule M(T);8:**return** the multicast tree *T* and the set of transmitting schedule M(T);


### 4.5. Approximation Ratio Analysis

In the following, we give the approximation ratio analysis of the proposed algorithm in Lemma 1 and Theorem 6 below.

**Lemma 1.**  Given a multicast request (s:D) in the duty-cycled network, the weighted sum of the minimum node-weighted Steiner tree is the lower bound for the MEMAP problem.

**Proof.**  Let $\left(T_{opt},M_{opt}\right)$ denote the optimal result for the MEMAP problem in the duty-cycled network *G*. Following the construction of the auxiliary graph $G^{\prime }$, $\left(T_{opt},M_{opt}\right)$ can be mapped into a node-weighted Steiner tree $T_{ag}^{\prime }$ in $G^{\prime }$, which is rooted at $s_{g}$ and spanning all of the nodes in $D_{g}$, and the transmitting schedule $\left(u,p,t\right)\in M_{opt}$ is mapped to a schedule node of $u_{s}$ in $T_{ag}^{\prime }$. Then, the total transmission power in $M_{opt}$ is equal to the weighted sum of the tree $T_{ag}^{\prime }$.

Assume $T_{ag}^{opt}$ is the minimum node-weighted Steiner tree in the auxiliary graph $G^{\prime }$, which spans all of the nodes in $D_{g}\cup \left\{s_{g}\right\}$. Obviously, we can get $W\left(T_{ag}^{opt}\right)\leq W\left(T_{ag}^{\prime }\right)$, where $W(T_{ag}^{opt})$ denotes the weighted sum of all of the nodes in $T_{ag}^{opt}$. The lemma is proven. ☐

**Theorem 6.**  *The approximation ratio of our method is* 4lnK, *where K is the number of the destination nodes.*

**Proof.**  Let $T_{ag}^{app}$ be the obtained approximate minimum node-weighted Steiner tree through [38]. According to [38], we have $W\left(T_{ag}^{app}\right)\leq 2lnKW\left(T_{ag}^{opt}\right)$, where $T_{ag}^{opt}$ is the minimum node-weighted Steiner tree.

Let $T_{ag}^{1}$, $T_{ag}^{2}$ and $T_{ag}^{3}$ denote the node-weighted Steiner tree after correcting Operations 1, 2 and 3, respectively. In the following, we will prove $W\left(T_{ag}^{3}\right)\leq 2W\left(T_{ag}^{app}\right)$.

Firstly, in correcting Operation 1, we remove the leaf nodes that does not belong to $D_{g}$; obviously, we can have:

(5) $$W\left(T_{ag}^{1}\right)\leq W\left(T_{ag}^{app}\right)$$

Secondly, in correcting Operation 2, we add the generation node of the schedule node $u_{s}$, of which the generation node is not in the tree *T*. Since the weight of all of the generation nodes is zero, we can have:

(6) $$W\left(T_{ag}^{2}\right)=W\left(T_{ag}^{1}\right)\leq W\left(T_{ag}^{app}\right)$$

Thirdly, in correcting Operation 3, let $u_{s}$ be the schedule node that is not derived from its father $i_{g}$. We handle this case in the following two aspects:

1. If we can find a schedule node $i_{s}\in ch\left(i_{g}\right)$ to reach the generation node $u_{g}$ of $u_{s}$, then we add an tree edge $\left(i_{s},u_{g}\right)$. No schedule node is added in this case. Thus, the weighted sum of the tree is not changed.
2. If we cannot find such a power, we need to add a schedule node $i_{s}^{\prime }$ with $i_{s}^{\prime }=argmin\left\{w\left(i_{s}\right)|i_{s}\in \Upsilon \left(i\right)⋀\left(i_{s},u_{g}\right)\in E^{\prime }\right\}$ in the correcting tree $T_{ag}^{2}$. Since we assume that the transmission is symmetric, so the weight of added schedule node $i_{s}^{\prime }$ is not larger than $w(u_{s})$.

Therefore, for each schedule node $u_{s}\in T_{ag}^{2}$, at most a schedule node with weight $w(u_{s})$ is added in the correcting tree $T_{ag}^{3}$. Therefore, we can have:

(7) $$W\left(T_{ag}^{3}\right)\leq 2W\left(T_{ag}^{2}\right)$$

According to Equations (5)–(7), we can have $W\left(T_{ag}^{3}\right)\leq 2W\left(T_{ag}^{app}\right)$. In addition, according to Lemma 1, we can have $W\left(T_{ag}^{app}\right)\leq 2lnKW\left(T_{ag}^{opt}\right)$ and $W\left(T_{ag}^{opt}\right)\leq W\left(T_{ag}^{\prime }\right)$, where $T_{ag}^{\prime }$ denotes the corresponding node-weighted Steiner tree for the optimal result for the MEMAP problem. Therefore, we can have $W\left(T_{ag}^{3}\right)\;\leq \;4lnKW\left(T_{ag}^{\prime }\right)$, and the approximation ratio is 4lnK. The theorem is proven. ☐

Additionally, the time complexity of the proposed algorithm can be proven to be polynomial by Lemma 2 and Theorem 7.

**Lemma 2.**  *The time complexity of Algorithm 1 is* $O(\Delta^{2})$.

**Proof.**  In Algorithm 1, since the number of nodes of $R(u_{s})$ is less than Δ, then Step 1 would take O(Δ×logΔ) time. In addition, we can see Steps 4–17 would take $O(|R\left(u_{s}\right)\left|\right)=O\left(\Delta \right)$ time obviously. Therefore, the time complexity from Steps 3–18 is $O(|R\left(u_{s}\right)|\times |R\left(u_{s}\right)\left|\right)=O\left(\Delta^{2}\right)$. Therefore, combining the above analysis, the time complexity of Algorithm 1 is $O(|R\left(u_{s}\right)|\times |R\left(u_{s}\right)|=O\left(\Delta^{2}\right)$. ☐

**Theorem 7.**  *The time complexity of the approximate scheduling and constructing algorithm is* $O(K^{2}L\Delta nlog\left(L\Delta n\right))$.

**Proof.**  Firstly, according to Definition 3, Step 1 would take O(Ln) time to create the schedule nodes and O(LΔn) time to create the edges. Then, the time complexity for Step 1 is O(LΔn). As for Step 2, there are O(Ln) schedule nodes, and the time complexity of Algorithm 1 is $\Delta^{2}$ according to Lemma 1, so the time complexity for Step 2 is $O(L\Delta^{2}n)$. In Step 3, since the number of nodes and edges are n+nLΔ and (1+Δ)×(nLΔ) according to Theorem 3, then the time complexity of Step 3 is $O(K^{2}L\Delta nlog\left(L\Delta n\right))$ according to [38]. In Step 4, we need to correct the obtained Steiner tree with three correction operations, where correction Operations 1 and 2 take O(LΔn) time by examining the tree once and correction Operation 3 would take O(LΔn) time by a breadth-first search. Therefore, the time complexity for Step 4 is O(LΔn). In Step 5, the time complexity is also O(LΔn) by visiting the corrected tree once. In summary, the time complexity of Algorithm 2 is $O(K^{2}L\Delta nlog\left(L\Delta n\right))$. ☐

## 5. Experimental Results

In this section, we study the empirical performance of the proposed algorithm. In the experiments, we randomly deploy the wireless nodes in a 300 m × 300 m field, where the number of nodes ranges from 100–200. The duty cycle is set from 5%–25%, and one time slot is set 50 ms. The working schedule of each node is generated randomly to test a wide range of configurations. In addition, each node in the experiments can transmit at five power levels, which are 1 MW, 10 MW, 15 MW, 20 MW and 50 MW respectively. The bandwidth is set to 40 kbps, and the size of the data is 100 bytes. All experiments are repeated 100 times with different node deployments and working schedules.

Since most existing methods are not suitable for minimum-energy multicasting with adjusted power in the duty-cycled sensor networks, we compare our algorithm (denoted as ASC) with the following baseline methods: the minimum spanning tree (MST) method and the shortest path tree (SPT) method. The SPT method calculates the shortest paths from the source node to each destination node, and then, all of the shortest paths form a multicast tree. As for the MST method, the leaf nodes that are not destination nodes are removed. This method is most widely used to approximate the Steiner tree. Additionally, in order to use the multicast tree constructed by the MST and SPT methods in the duty-cycled sensor networks, the transmitting schedules, including the transmitting time slot and the transmitting power for each non-leaf node, should also be determined. In the experiments, we exploit the enumerating method to find the best transmitting schedules for the multicast tree constructed by the MST and SPT methods.

### 5.1. Performance of Energy Cost

**Figure 3:**
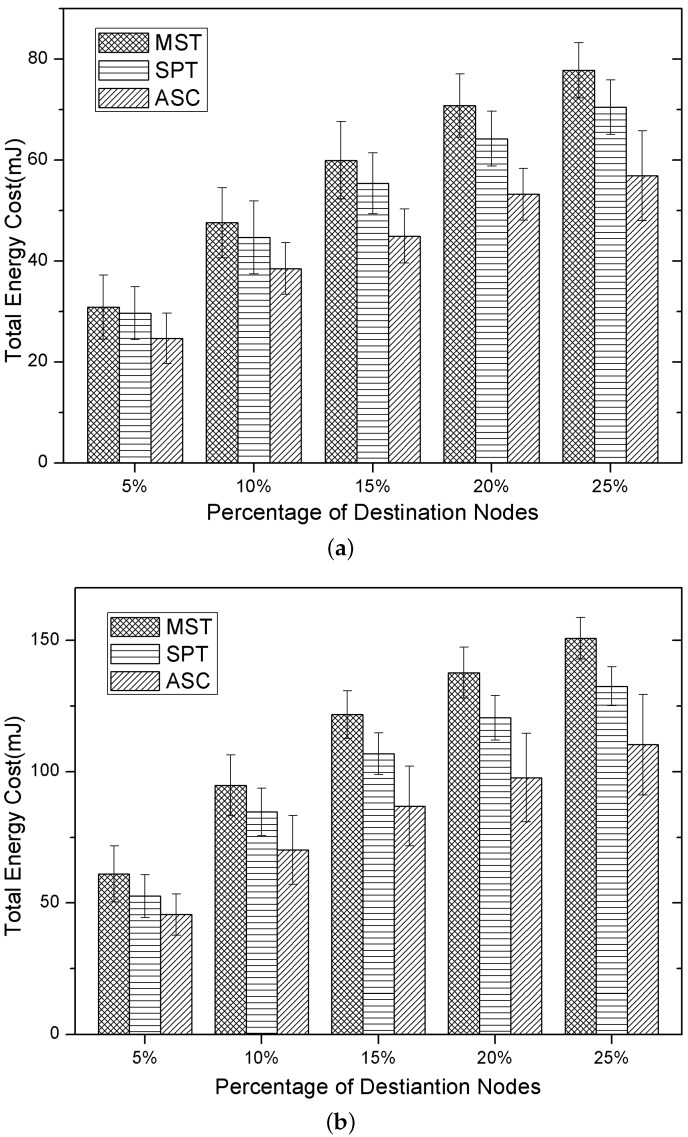
Total energy cost. (**a**) |V| = 100; (**b**) |V| = 200.

Firstly, we compare the total energy cost of three algorithms under different numbers of destination nodes. We conduct the experiments mainly in two network scenarios, while the number of nodes is set to 100 and 200, respectively, and the results are shown in Figure 3a,b. In Figure 3a, the x-coordinate denotes the percentage of the destination nodes of all nodes, which ranges from 5%–25%. As we can see, the total energy cost produced by our method is the lowest, which is about 20% lower than the MST method, and when the number of destination nodes is greater, the ratio can even reach 30%. This is because our method considers the working schedule and the transmitting power of each node in constructing the multicast tree, which can reduce the energy cost and the number of transmissions at the same time. Although the MST and SPT method determine the transmission schedule optimally in a localized way, they construct the multicast tree regardless of the node’s working schedule and cannot optimize the multicast tree in a global manner. It’s to be noticed that in Figure 3a, the total energy cost of the SPT method is a little lower than the MST method; this is because the SPT method reduces the number of transmissions through adjusting the transmitting power. In Figure 3b, the total energy cost of three methods is increased compared to Figure 3a; this is because the number of nodes and the destination nodes increased. However, our algorithm still generates the lowest energy cost compared to the other two methods. Additionally, we can see that the total energy cost of the three algorithms grows with the number of destination nodes increasing in both experiments.

### 5.2. Performance of Total Transmissions

**Figure 4:**
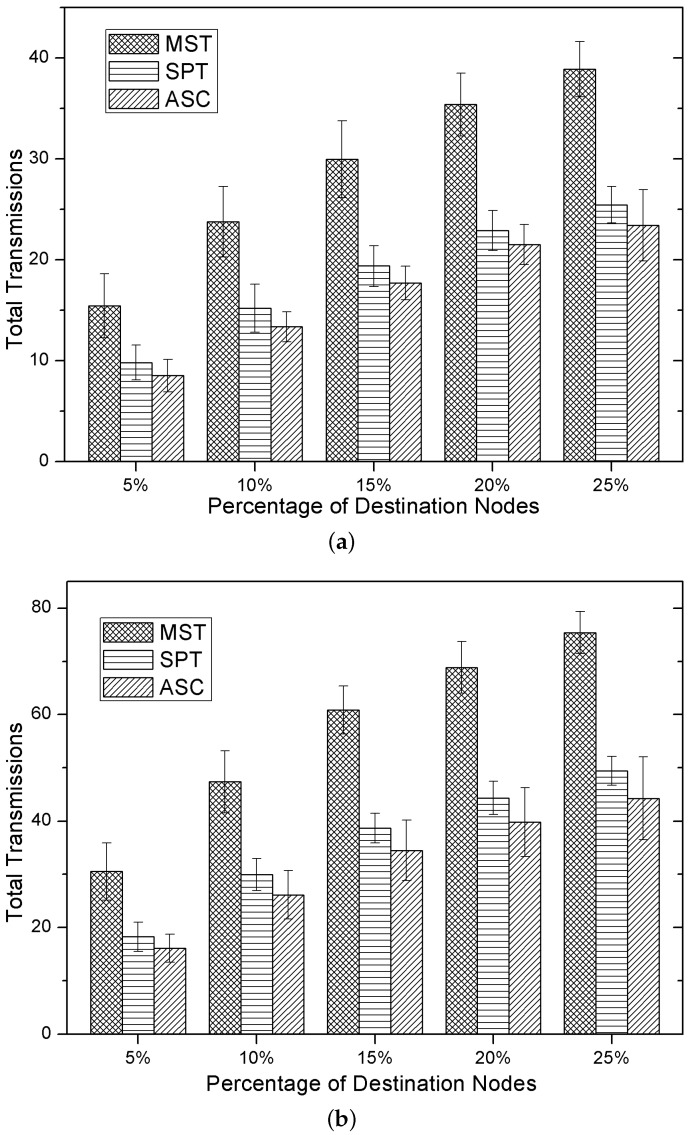
The total number of transmissions. (**a**) |V| = 100; (**b**) |V| = 200.

In this group of experiments, we analyzed the total number of transmissions of three methods in the multicasting process. In the duty-cycled sensor networks, besides the transmitting power of all of the nodes in the multicast tree, the total number of transmissions is also an important factor for the total energy cost. As shown in Figure 4a,b, the total number of transmissions of the the SPT method is far less than the MST method. This results in that the energy cost of the SPT method is less than the MST method (which has been demonstrated in Figure 3a,b), despite that the SPT method exploits higher transmitting power in multicasting. However, both the total number of transmissions of the MST method and the SPT method are larger than our method. Additionally, as shown in Figure 4a,b, the total number of transmissions of three methods increases with the number of destination nodes.

### 5.3. Performance under Different Duty Cycles

**Figure 5:**
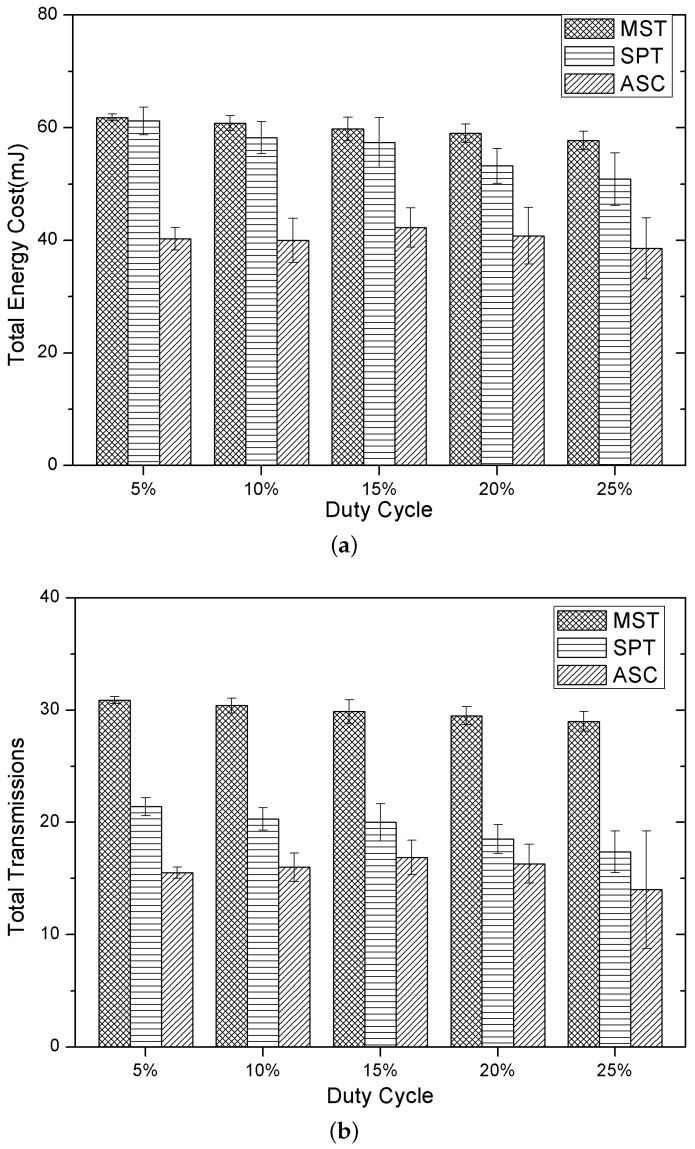
Performance under different duty cycles. (**a**) Total energy cost; (**b**) Total transmissions.

Finally, we investigate the influences of different duty cycles on the performance of the three methods, and the results are shown in Figure 5a,b. In this group of experiments, the duty cycle of each node varies from 5%–20%, and the percentage of the destination node is set as 15%. As we can see in Figure 5a,b, the total energy cost and the number of transmissions of our method are both the lowest, which demonstrates the high performance of our method in terms of energy cost. Additionally, in Figure 5a, we can find that the total energy cost of our method decreases slightly with the duty cycle increasing. This may be because our method has exploited the working schedule of each node in constructing the multicasting tree, and the common active slots of the neighboring nodes do not vary much when the duty cycle increases 5%, which results in the total number of transmissions not reducing obviously. This can be illustrated in Figure 5b, where the number of transmissions of the three methods decrease slightly when the duty cycle of each node increases 5%.

## 6. Conclusions

In this paper, the problem of minimum energy multicasting with adjusted power (MEMAP) in duty-cycled sensor networks was proposed, and it was proven to be NP-hard. To solve such a problem, an auxiliary graph was proposed to integrate the transmitting power and time slot scheduling problem and the minimum multicast tree constructing problem in MEMAP, and a greedy algorithm was exploited to construct such a graph. Based on the proposed auxiliary graph, an approximate scheduling and constructing algorithm with an approximation ratio of 4lnK was proposed, where *K* is the number of destination nodes. Finally, we perform extensive simulations, and the results verify the high performance of the proposed algorithm in terms of the energy cost and transmission redundancy.
